# *Vibrio natriegens* is sensitive to its acidic fermentation products

**DOI:** 10.1128/aem.01745-25

**Published:** 2026-02-09

**Authors:** Nicholas W. Haas, James B. McKinlay

**Affiliations:** 1 Department of Biology, Indiana University 123993, Bloomington, Indiana, USA; Danmarks Tekniske Universitet, Kgs. Lyngby, Denmark

**Keywords:** lactate dehydrogenase, microbial physiology, industrial microbiology, stress response, acid tolerance, synthetic biology, fermentation, *Vibrio natriegens*, RpoS

## Abstract

*Vibrio natriegens* is an emerging bacterial platform for a range of biotechnological applications due to its rapid growth and ease of genetic manipulation. Whereas much has been learned about *V. natriegens’* aerobic physiology, comparatively little is known about its anaerobic fermentative physiology, despite its relevance to many industrial conditions. We compared the metabolic parameters of *V. natriegens* versus another biotechnologically relevant bacterium, *Escherichia coli,* under fermentative conditions. Both species excreted a similar array of fermentation products, but *V. natriegens* consumed less glucose and had a lower product titer. *V. natriegens* also exhibited rapid death, reaching extinction within 12 h after the growth phase, 3 days sooner than *E. coli*. Rapid *V. natriegens* death was avoided, and glucose consumption and product titers improved, by increasing the buffering capacity of the growth medium, indicating that *V. natriegens* is comparatively sensitive to its organic acid fermentation products. Aside from a minor role for RpoS in acid resistance, the *V. natriegens* genome lacks nearly all the acid resistance genes that have been characterized in *E. coli* and *Vibrio cholerae*. Our findings thus highlight an acid sensitivity that will need to be considered when designing fermentative applications of *V. natriegens*.

**IMPORTANCE:**

Bioprocessing, the biological conversion of renewable resources into value-added chemicals, is poised to meet an increasing demand for sustainable alternatives to petroleum-based products. Many examples of bioprocessing feature anoxic fermentations that naturally maximize product formation relative to growth of the microbial catalyst. *Vibrio natriegens* is a facultatively fermentative bacterium that has gained attention for bioprocessing due to its rapid growth rate and ease of genetic engineering. However, the fermentative properties of *V. natriegens* have not been compared to traditional bioprocessing workhorses like *Escherichia coli*. We revealed that *V. natriegens* is comparatively sensitive to its own acidic fermentation products, likely because *V. natriegens* lacks acid resistance mechanisms possessed by *E. coli*. Thus, fermentative applications must address this sensitivity either by buffering the fermentations, engineering resistance mechanisms, or bypassing the sensitivity by engineering *V. natriegens* to produce neutral products.

## INTRODUCTION

Bioprocessing utilizes native or engineered microbes to generate value-added products from renewable resources, offering sustainable alternatives to petroleum-based counterparts (1–5). A diverse set of microbes has been proposed as platforms, or chassis, for bioprocessing (6–8). The marine bacterium *Vibrio natriegens* has increasingly gained attention as a chassis for bioprocessing primarily due to its rapid growth and metabolism (9–11) and the relative ease with which combinatorial genetics can be performed through natural transformation (12–15). Proof-of-concept studies have demonstrated that *V. natriegens* can be engineered to produce a variety of value-added products from a range of carbon substrates (11). However, whereas many bioprocesses use anoxic fermentative conditions to maximize product yields and minimize microbial growth, most *V. natriegens* studies have used oxic conditions, with a few exceptions (10, 16, 17).

**Fig 1:**
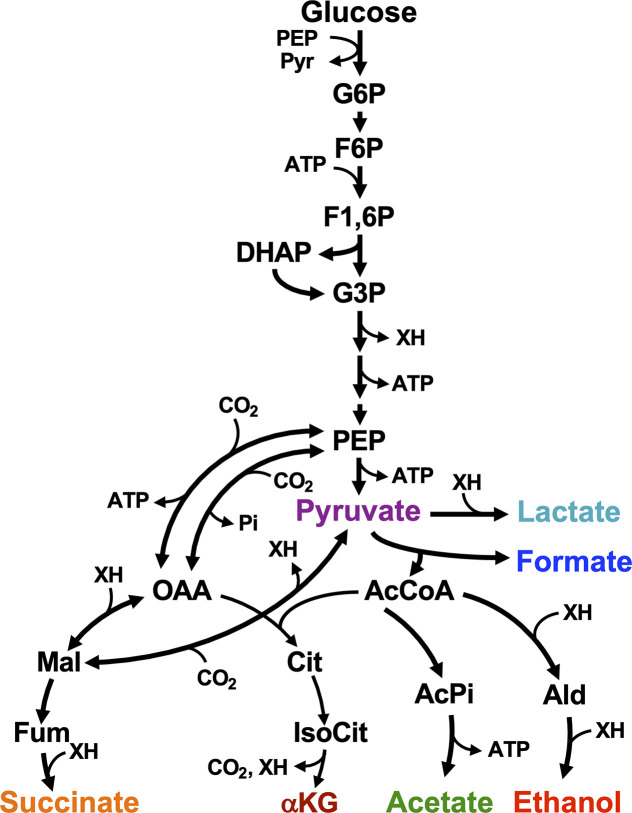
*V. natriegens* fermentative metabolism. Reactions are based on NCBI gene annotations. Acidic products are named here for their carboxylate forms. XH, electron carriers [e.g., NAD(P)H].

Under anoxic conditions with glucose, *V. natriegens* carries out a mixed-acid fermentation, excreting ethanol and a variety of organic acids (Fig. 1) (10). The accumulation of organic acids acidifies the medium. In general, acid stress negatively impacts metabolism and viability. Negative impacts of an acidic environment are amplified when the low pH favors protonation of excreted organic acids, allowing them to diffuse into the cytoplasm where they then dissociate, acidifying the cytoplasm, weakening the proton motive force, and increasing the cytoplasmic anion concentration (18, 19). Even under aerobic conditions, *V. natriegens* growth is impacted by acidification from acetic acid that accumulates due to overflow metabolism (20). Thus, it is expected that these negative impacts would be exacerbated under anoxic fermentative conditions where there is a greater accumulation of organic acids. Understanding how *V. natriegens* responds to its fermentation products is important for designing future bioprocesses, as organic acids affect important production parameters including rate, fermentation time, yield, and associated costs for neutralization and waste treatment (21–23).

Whereas other *Vibrio*s like *V. cholerae* can switch to neutral fermentation products in response to acidic conditions (24), production of neutral fermentation products by *V. natriegens* has not been observed (10). Fermentative bacteria like *Escherichia coli* have other mechanisms to deal with acid stress (23, 25). For example, *E. coli* can offset acidosis by converting formic acid into H_2_ and CO_2_ gases using formate hydrogen lyase (FHL) (26). In the presence of certain amino acids, *E. coli* and *V. cholerae* can also eliminate cytoplasmic protons via amino acid decarboxylation reactions (27, 28). As the medium acidifies during fermentation, *E. coli* also shifts its fermentation profile towards lactic acid (29), which might also offset acidosis by producing a single acid instead of the combination of acetic and formic acid (30) and avoiding accumulation of intracellular acetate anions (31). *E. coli* also has different global regulatory responses to organic acids like acetate, formate, and lactate (30, 32–34). The extent to which *V. natriegens* can use these or other acid resistance mechanisms during mixed acid fermentation is unknown.

Here, we compared the growth and metabolic parameters of *V. natriegens* versus *E. coli* under anoxic fermentative conditions. Whereas both organisms produced similar fermentation products, *E. coli* cultures consumed more glucose, accumulated more organic acids, and exhibited longer stationary-phase viability. We conclude that *V. natriegens* is comparatively sensitive to fermentative organic acids due to a lack of acid resistance mechanisms.

## RESULTS AND DISCUSSION

### *V. natriegens* and *E. coli* have similar fermentative product yields

**Fig 2:**
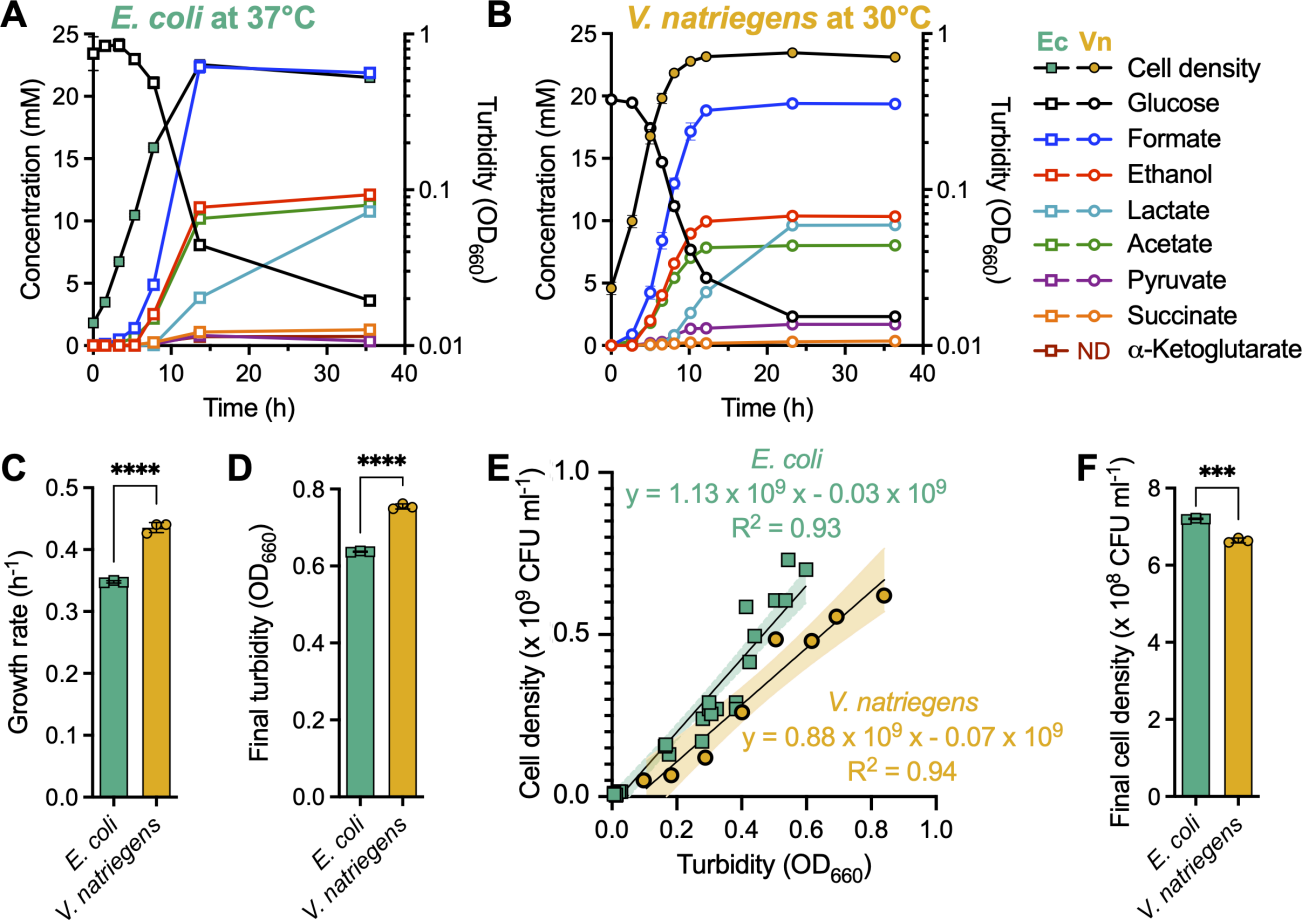
*V. natriegens* grows faster but to a lower cell density while producing a similar array of fermentation products as *E. coli. E. coli* (**A**) and *V. natriegens* (**B**) were grown in 60 mL of anoxic minimal media at 37°C or 30°C, respectively, and growth, glucose, and fermentation products were monitored. The same cultures were used to determine exponential growth rate (**C**) and final (highest) cell density (**D**). Standard curves to convert turbidity (OD_660_) measurements of cell density to CFU mL^−1^ (**E**) suggest that the difference in final cell densities between the two species is opposite when considering turbidity (**D**) versus CFU mL^−1^ (**F**). (**C, D, F**) Points, biological replicate values; bars, mean; error bars, SD; *n* = 3. Statistical differences were determined using an unpaired, two-tailed *t*-test; ***, *P* < 0.001; ****, *P* < 0.0001. (**E**) Points, biological replicate values; lines, fitting from simple linear regression analysis; shading, SD.

*V. natriegens* is often compared to *E. coli*, a bacterium that is well-established in synthetic biology, as a potential platform for bioprocessing (9). However, a direct comparison of growth and metabolic parameters under fermentative conditions has not been reported. We grew *E. coli* MG1655 and *V. natriegens* ATCC14048 in their respective anoxic minimal media with 25 mM glucose as the sole carbon source. The media differed by a higher NaCl concentration for *V. natriegens* and by temperature; 37°C was used for *E. coli* versus 30°C for *V. natriegens*. Although *V. natriegens* was originally reported to grow ~1.06 times faster at 37°C (35), several groups grow *V. natriegens* at 30°C (12, 36–38), including in comparisons to *E. coli* where advantages have been noted at 30°C for *V. natriegens* protein production (39–42). Despite the lower growth temperature, *V. natriegens* grew 1.3 times faster than *E. coli* (Fig. 2A through C). Although *V. natriegens* reached a higher turbidity (OD_660_; Fig. 2D), *E. coli* has a higher colony-forming units (CFU) mL^−1^:OD_660_ ratio (Fig. 2E) and was thus estimated to achieve a 1.2-fold higher actual cell density (CFU mL^−1^) than *V. natriegens* (Fig. 2F).

**Fig 3:**
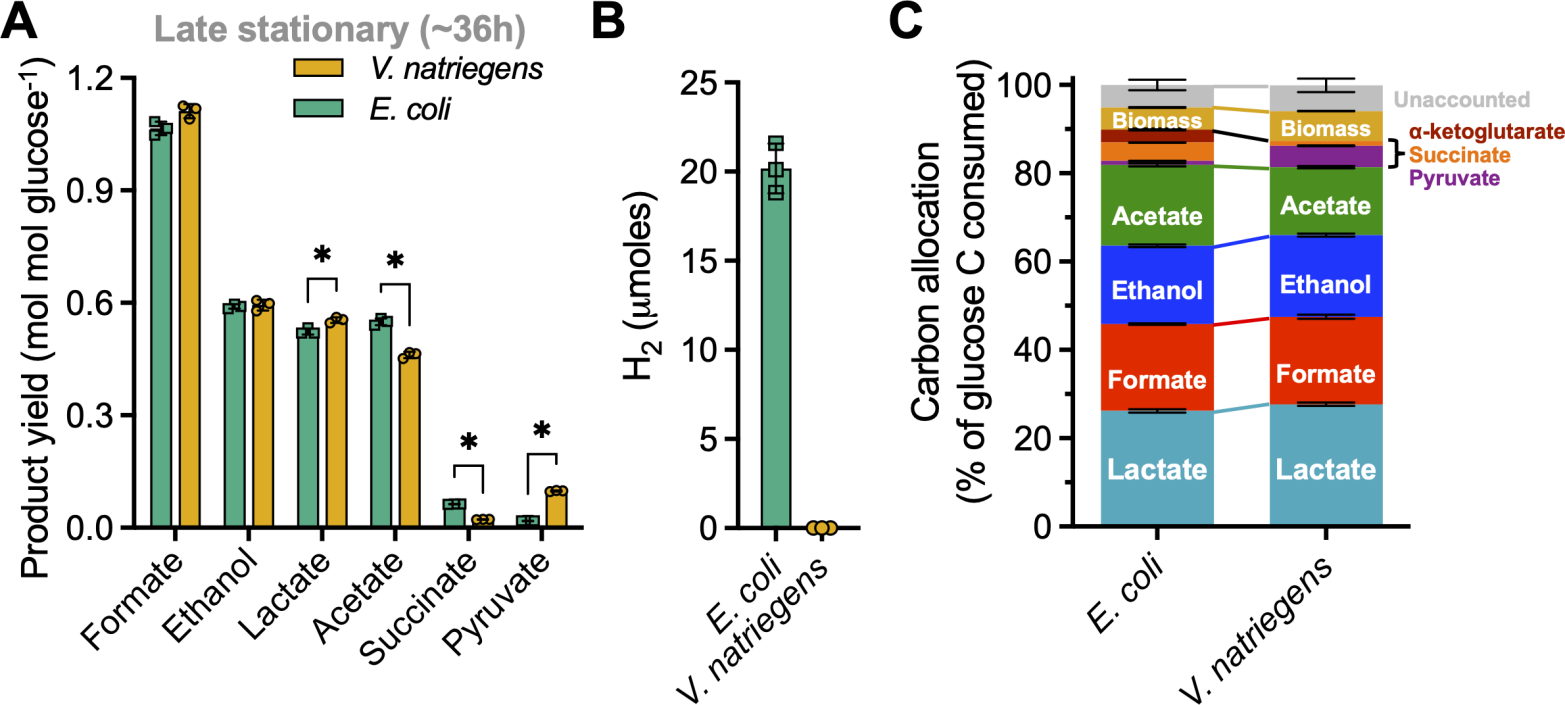
*V. natriegens* and *E. coli* major soluble fermentation product profiles are similar. Fermentation product yields (**A**), H_2_ accumulated in the headspace (limit of detection ~10 nmol) (**B**), and carbon allocations (**C**) were determined using late stationary-phase (36 h) samples. All samples were from the same cultures as for Fig. 2 except for H_2_. (**A–C**) Points, biological replicate values; bars, mean; error bars, SD; *n* = 3. (**A**) Statistical differences were determined using an unpaired, two-tailed *t*-test; *, *P* < 0.05. For formic acid; EtOH, ethanol; Lac, lactic acid; Ace, acetic acid; Suc, succinic acid; Pyr, pyruvate. (**C**) Turbidity measurements were converted into moles of biomass carbon (**C**) as described in Materials and Methods.

Late stationary-phase (36 h) fermentation product yields between *E. coli* and *V. natriegens* were either statistically similar or exhibited small differences for the most abundant fermentation products: formic acid, no difference; ethanol, no difference; lactic acid, 1.06-fold higher for *V. natriegens*; acetic acid, 1.20-fold higher for *E. coli* (Fig. 3A). Both species produced lactic acid as they exited exponential phase, with continued production into stationary phase (Fig. 2A and B). Greater fold differences were observed for minor fermentation product concentrations (Fig. 2A and B) and yields (Fig. 3A): (i) *E. coli* produced ~1.3 mM succinic acid whereas levels from *V. natriegens* were near the detection limit, (ii) *V. natriegens* produced ~1.7 mM pyruvate that was stable in the supernatant whereas *E. coli* transiently produced as much as 0.8 mM pyruvate, and (iii) *E. coli* produced ~0.8 mM α-ketoglutarate, whereas none was detected in *V. natriegens* supernatants (Fig. 2A and B). Another key difference was that *V. natriegens* did not produce H_2_, whereas *E. coli* is known to produce H_2_ via FHL as the pH drops below neutral (Fig. 3B) (43, 44); the *V. natriegens* genome does not encode FHL nor any hydrogenase (not shown). A carbon balance analysis revealed that only 5 ± 1% (*E. coli*) and 6 ± 2% (*V. natriegens*) of carbon was unaccounted for outside of fermentation products and biomass (Fig. 2C). Missing carbon could be explained in part by amino acids; Hoffart et al. found that extracellular alanine, valine, and glutamate could account for 4% of glucose carbon consumed by *V. natriegens* under fermentative conditions (10). Below, we focus on the major organic acid fermentation products.

### *V. natriegens* is more sensitive to acid than *E. coli*

**Fig 4:**
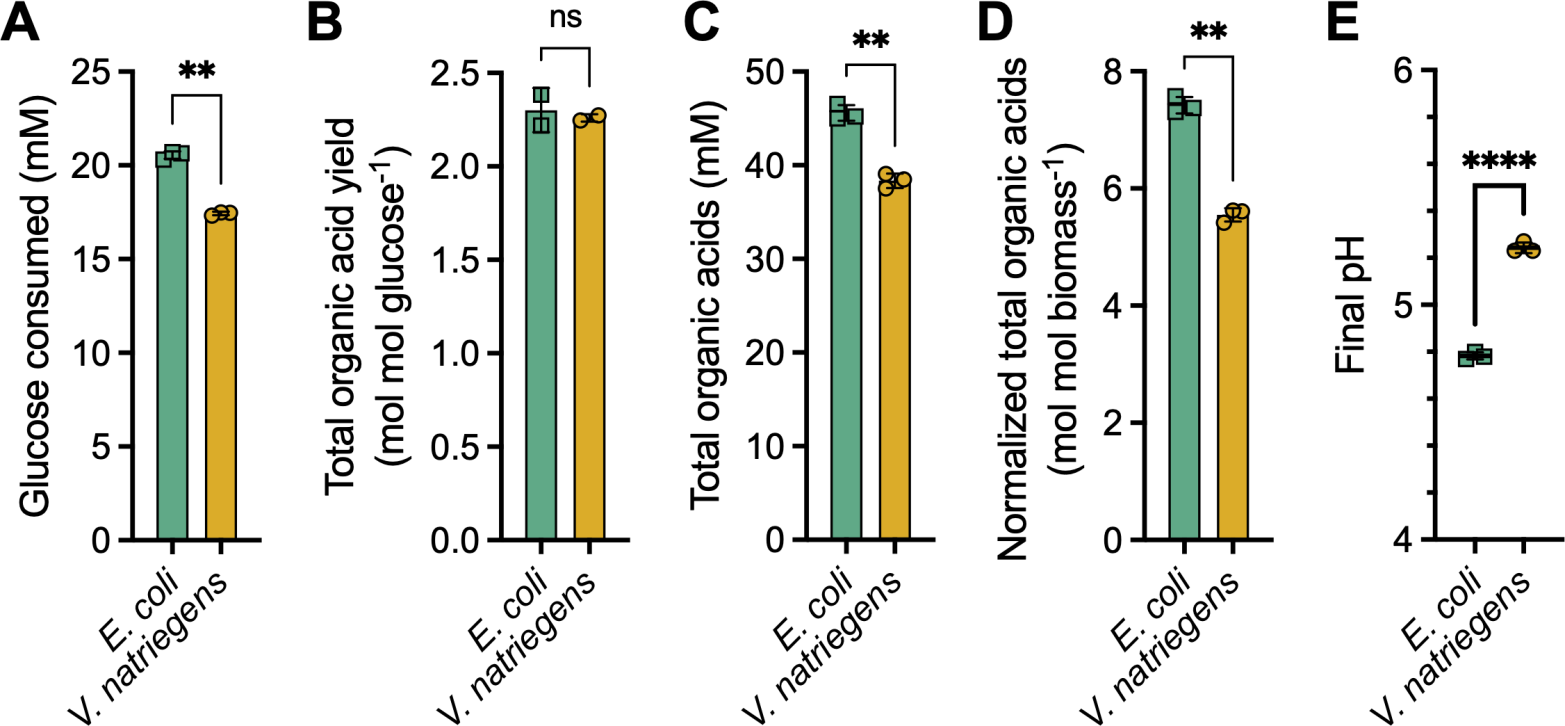
*V. natriegens* consumes less glucose and produces less organic acid than *E. coli*. Glucose consumed (**A**), total organic acid yield (**B**), total organic acid concentration (**C**), organic acid concentration normalized for biomass carbon (**D**), and supernatant pH (**E**) were determined using late stationary-phase samples (36 h, except for pH, taken at 24 h). All samples were from the same cultures as for Fig. 2 except for pH measurements. Points, biological replicate values; bars, mean; error bars, SD; *n* = 3. Statistical differences were determined using an unpaired, two-tailed *t*-test; ns, not significant; **, *P* < 0.01; ****, *P* < 0.0001. (**D**) Turbidity measurements were converted into moles of biomass carbon (**C**) as described in Materials and Methods.

Neither strain fully consumed the glucose provided (Fig. 2A and B) but *E. coli* consumed 1.2 times more glucose than *V. natriegens* (Fig. 4A). A possible reason is that each species reached its acid tolerance threshold, and *V. natriegens* has a lower acid tolerance. Indeed, although each species had a similar total organic acid yield (Fig. 4B), *E. coli* accumulated 1.2-fold more organic acids (Fig. 4C) and 1.3-fold more organic acids per cell (Fig. 4D). As a result, the final *E. coli* pH was more acidic at 4.8 ± 0.0 compared to 5.2 ± 0.0 for *V. natriegens* (Fig. 4E).

**Fig 5:**
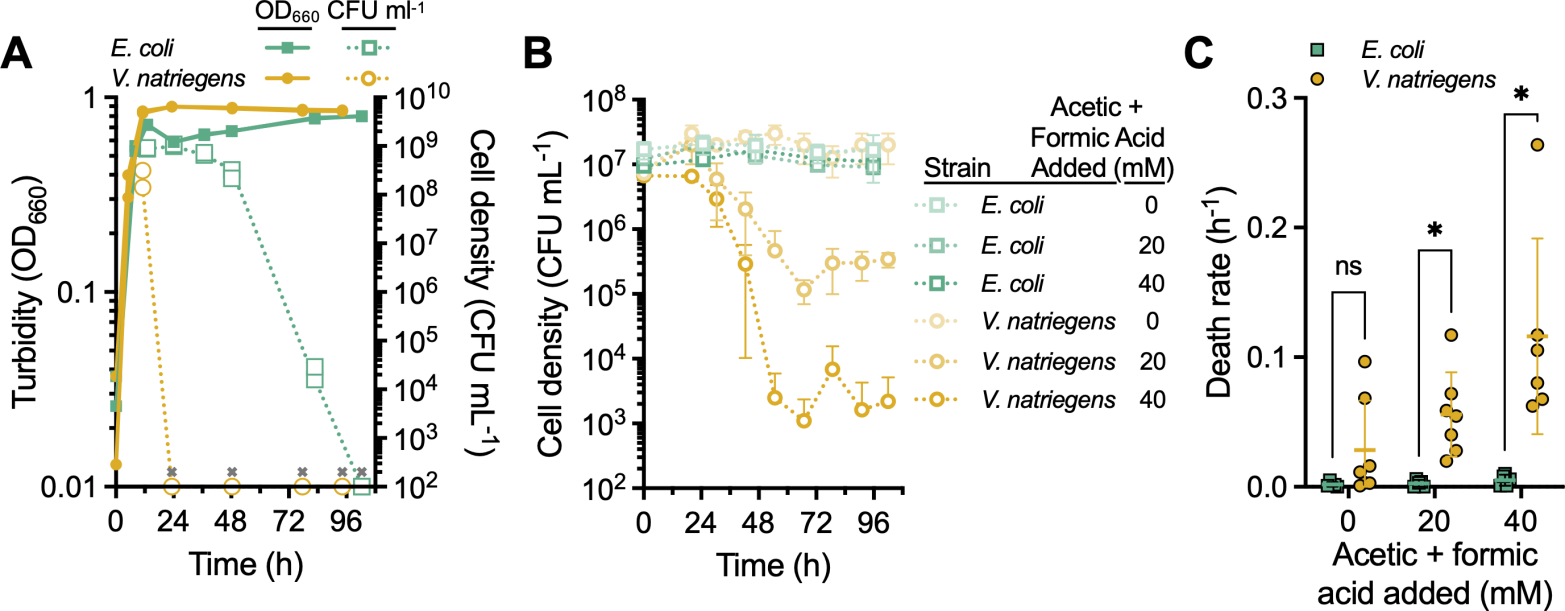
*V. natriegens* is sensitive to acidic fermentation products compared to *E. coli*. (**A**) Culture turbidity (solid lines; OD_660_) was tracked in minimal media with 25 mM glucose over the entire experiment. Viable cell density (dotted lines; CFU mL^−1^) was tracked from the end of the growth phase (~12 h). All data points from the duplicate cultures are shown. Symbols with an “x” were below the detection limit of 10^2^ CFU mL^−1^. (**B**) Viable cell counts were monitored in cell suspensions inoculated in late exponential phase to defined media lacking glucose with different concentrations of a 1:1 solution of acetic and formic acid. Data points, mean; error bars, SD; *n* = 3. (**C**) Death rates determined from the slope of a semi-log plot fitted to the data from panel B and two other similar experiments that differed by starting cell densities (range: 0.05–2.0 × 10^7^ CFU mL^−1^) and incubation time (range: 140–155 h). Horizontal line, mean; error bars, SD; *n* = 5–7. Statistical differences were determined using unpaired, two-tailed *t*-tests; ns, not significant; *, *P* < 0.05. (**A, B**) The detection limit is 10^2^ CFU mL^−1^.

To compare the acid tolerance between the two species, we tracked cell viability by CFUs during stationary phase (Fig. 5A). Herein, we assume that a decline in CFUs is equivalent to death, especially given the acidic conditions, but we acknowledge that a viable but non-culturable (VBNC) state is also possible, as has been described for other Vibrios (45). However, whereas the VBNC state is important for epidemiology, VBNC cells are of no practical use for value-added chemical production due to their lack of metabolic activity. Although death was not evident from turbidity measurements, CFU measurements indicated that both species ultimately went extinct. However, whereas viable *E. coli* cells were detected up to 82 h after inoculation, no viable *V. natriegens* cells were detected 24 h after inoculation (limit of detection = 10^2^ CFU mL^−1^), about 12 h after the end of the exponential growth phase (Fig. 4A).

To verify that organic acids were a primary driver of *V. natriegens* cell death, we inoculated late exponential phase cultures (*V. natriegens*, 0.65–0.69 OD_660_; *E. coli*, 0.82–0.87 OD_660_) to media lacking glucose but with different concentrations of a 1:1 mixture of acetic and formic acid. The cultures used to prepare these suspensions were grown with less glucose (15 mM) to ensure less acid exposure prior to the assay. Without added organic acids, both species showed little decline in cell viability (Fig. 5B and C). *E. coli* viability was also unaffected by 20 and 40 mM organic acids (Fig. 5B and C). In contrast, increasing the organic acid concentration progressively decreased *V. natriegens* viability (Fig. 5B) and increased the death rate (Fig. 5C).

**Fig 6:**
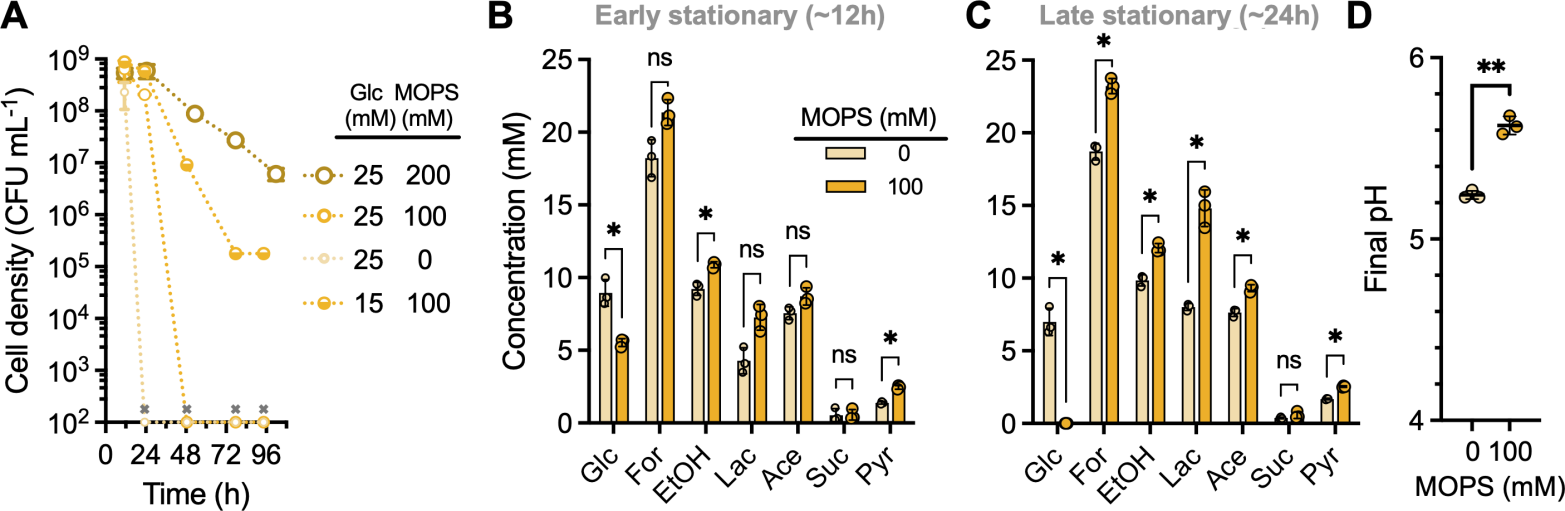
Increasing the buffer concentration of the growth medium alleviates *V. natriegens* organic acid sensitivity. (**A**) *V. natriegens* viable cell density was tracked from the end of the growth phase (~12 h) in minimal media with different concentrations of glucose and MOPS (pH 7). The “0 mM MOPS” data are the same as in Fig. 5A. Data points, mean; error bars, range; *n* = 2; some error bars are smaller than the data point symbols. Symbols with an “x” were below the detection limit of 10^2^ CFU mL^−1^. (**B, C**) Remaining glucose and accumulated fermentation products from *V. natriegens* cultures grown with 25 mM glucose in early stationary phase (B, ~ 12 h) and late stationary phase (C, ~ 24 h) with or without 100 mM MOPS (pH 7). Glc, glucose; For, formic acid; EtOH, ethanol; Lac, lactic acid; Ace, acetic acid; Suc, succinic acid; Pyr, pyruvate. (**D**) Late stationary-phase (24 h) pH values from cultures grown with 25 mM glucose with or without 100 mM MOPS (pH 7). Values without MOPS are the same as those in Fig. 4E. (**B–D**) Points, biological replicate values; bars, mean; error bars, SD; *n* = 3. Statistical differences were determined using unpaired, two-tailed *t*-tests; ns, not significant; *, *P* < 0.05; **, *P* < 0.01.

We then verified that the *V. natriegens* sensitivity to organic acids is dependent on the pH by increasing the buffering capacity of medium with either 100 or 200 mM 4-morpholinepropanesulfonic acid (MOPS) buffer. In each case, viable cell counts exceeded 10^8^ CFU mL^−1^ at the onset of stationary phase (Fig. 6A). Unlike the rapid extinction observed when MOPS was excluded, 100 mM MOPS delayed extinction by 24 h and 200 mM MOPS avoided extinction within the 100 h time course (Fig. 6A). Even though cells still went extinct with 100 mM MOPS, glucose was fully consumed by 24 h, with continued production of most fermentation products, especially lactic acid (Fig. 6B and C). The final pH was also correspondingly higher when 100 mM MOPS was provided, as expected (Fig. 6D). Thus, we conclude that the rapid extinction of *V. natriegens* upon reaching stationary phase under fermentative cultures is due to acid sensitivity.  

### *V. natriegens* lacks acid resistance mechanisms

We sought an explanation for the stark difference between *V. natriegens* and *E. coli* survival in acidic conditions. Bacteria like *E. coli* and some well-characterized *Vibrios*, like *V. cholerae,* have mechanisms to deal with acid stress both during fermentative conditions and to transit the highly acidic stomach (23, 25, 28, 46). To determine the acid resistance repertoire of *V. natriegens*, we performed BLASTp searches (47) using known *E. coli* and *V. cholerae* acid resistance proteins as query sequences (Table 1). We focused on mechanisms involving cell envelope stress and specific acid response mechanisms. We excluded other proteins that emerge in functional genomic surveys but are commonly used by most bacteria across diverse conditions (30, 32–34, 48).

**TABLE 1:** Inventory of possible *V. natriegens* acid tolerance proteins

			*V. natriegens* homolog			
Gene	Description	Query NCBI RefSeq	Coverage (%)	Identity (%)	Locus tag	Protein ID
Cell envelope/general stress response						
*rpoE*	σE/σ24/RpoE	NP_417068/b2573	100	75	PN96_RS01020	WP_014233001
*rseA*	Anti-σE	NP_417067/b2572	96	41	PN96_RS01025	WP_014233000
*rseB*	Anti-σE stabilizer	NP_417066/b2571	96	44	PN96_RS01030	WP_014232999
*rseC*	Regulatory protein	NP_417065/b2570	81	34	PN96_RS01035	WP_014232998
*cpxR*	Transcriptional regulator	NP_418348/b3912	98	61	PN96_RS14495	WP_020336210
*cpxA*	Sensor histidine kinase	NP_418347/b3911	98	44	PN96_RS14490	WP_020336211
*cpxP*	Periplasmic stress protein	YP_026277/b4484	71	31	PN96_RS14500	WP_020336209
*cfa*	Cyclopropane FA synthase	NP_416178/b1661	90	37	PN96_RS07970	WP_020332938
*hdeABD*	Periplasmic chaperones	NP_417966-8/b3509-11	No homolog			
*skp*	Periplasmic chaperone	NP_414720/b0178	No homolog			
*degP*	Periplasmic endoprotease	NP_414703/b0161	No homolog			
*rffG*	LPS modification	YP_026255/b3788	96	55	PN96_RS12400	WP_020335757
*rffH*	LPS modification	NP_418236/b3789	98	63	PN96_RS12375	WP_020335762
*toxR*	Transcriptional regulator	WP_000018139	100	53	PN96_RS09525	WP_014231125
*ompU*	Porin	WP_001044323	100	65	PN96_RS01620	WP_020333911
*vc1080*	Phosphotransferase	WP_000438236	No homolog			
FHL						
*fhlA*	Transcriptional regulator	NP_417211/b2731	47	44	PN96_RS02620	WP_020333608
*hycA*	Regulator of FhlA	NP_417205/b2725	No homolog			
*hycB*	FHL subunit B	NP_417204/b2724	93	29	PN96_RS05940	WP_014231815
*hycC*	FHL subunit C	NP_417203/b2723	55	27	PN96_RS21860	WP_020336034
*hycDE*	FHL subunit D, E	NP_417201,2/b2721,2	No homolog			
*hycF*	FHL subunit F	NP_417200/b2720	67	29	PN96_RS05960	WP_014231811
*hycGHI*	FHL subunit G, assembly	NP_417197-9/b2717-9	No homolog			
*hypA*	Hydrogenase Ni incorporation	NP_417206/b2726	No homolog			
*hypB*	Hydrogenase Ni incorporation	NP_417207/b2727	25	26	PN96_RS01805	WP_014232860
*hypCDE*	Hydrogenase maturation	NP_417208-10/b2728-30	No homolog			
Amino acid decarboxylases/ ion balance						
*gadA*	Glutamate decarboxylase A	NP_417974/b3517	No homolog			
*gadB*	Glutamate decarboxylase B	NP_416010/b1493	No homolog			
*gadC*	GABA antiporter	NP_416009/b1492	No homolog			
*rpoS*	σS/σ38/RpoS	NP_417221/b2741	96 (E71*)^*a*^	75	PN96_RS01145	WP_061778639
*adiA*	Arginine decarboxylase	NP_418541/b4117	No homolog			
*adiC*	Arginine:agmatine antiporter	NP_418539/b4115	96	27	PN96_RS21655	WP_020335996
*cadA*	Lysine decarboxylase	NP_418555/b4131	No homolog			
*cadB*	Lysine:cadaverine antiporter	NP_418556/b4132	72	26	PN96_RS18150	WP_014233759
*speC*	Ornithine decarboxylase	NP_417440/b2965	No homolog			
*speF*	Ornithine decarboxylase	NP_415220/b0693	No homolog			
*clcA*	H^+^/Cl^-^ antiporter	NP_414697/b0155	93	56	PN96_RS18465	WP_014233820
*clcB*	H^+^/Cl^-^ antiporter	NP_416109/b1592	No homolog			
*trkG*	Na^+^/K^+^ antiporter	NP_41588/b1363	99	42	PN96_RS13405	WP_014230441
*trkH*	K^+^ channel	YP_026273/b3849	100	69	PN96_RS13405	WP_014230441
*kdpA*	K^+^ transporter (ATP)	NP_415226/b0698	No homolog			
*kdpB*	K^+^ transporter (ATP)	NP_415225/b0697	75	29	PN96_RS09780	WP_020333332
*kdpC*	K^+^ transporter (ATP)	NP_415224/b0696	No homolog			
Acetoin and 2,3-butanediol production						
*budA*	Acetolactate decarboxylase	WP_001068896	No homolog			
*alsO*	Acetoin reductase	WP_001214790	Multiple SDR oxidoreductase hits			
Small proteins						
*yqgB*	Acid stress small protein	NP_417414/b2939	No homolog			
*mgrB*	Acid stress small protein	NP_416340/b1826	No homolog			
*yobF*	Acid stress small protein	NP_416338/b1824	No homolog			
*yceO*	Acid stress small protein	NP_415576/b1058	No homolog			

^*a*^ RpoS in our strain has an E71* mutation.

Based on our homolog threshold criteria of 25% identity over 50% of the query sequence, *V. natriegens* likely has several proteins to respond to cell envelope stress, which could include acid stress, but also other stressors, like heat. For example, *V. natriegens* has proteins with 53 and 65% amino acid identity to the *V. cholerae* transcriptional regulator ToxR and porin OmpU, respectively. These proteins are involved in *V. cholerae* tolerance to organic acids at low pH but can also respond to other stressors (46). *V. natriegens* also has proteins with 55 and 63% amino acid identity to the *E. coli* LPS-modification enzymes that were demonstrated to improve acid tolerance (48). We also identified a σ^E^ homolog and associated proteins that, in *E. coli*, activate a regulon in response to denatured outer membrane proteins, and the Cpx two-component system that responds to periplasmic and inner membrane stress signals (49). Among genes of the *E. coli* Cpx regulon, *V. natriegens* also has *cfa*, which encodes a protein that produces cyclopropane fatty acids that can protect against various stressors, including acidity (50). However, *V. natriegens* appears to lack many of the chaperones and proteases (i.e., *degP*, *hdeABD*, *skp*) that are crucial in *E. coli* for the proper folding or degradation of cell envelope proteins under stress conditions (51–53).

When considering specific responses to acid stress, the *V. natriegens* genome suggests that it is even less well-equipped. FHL, a complex of formate dehydrogenase (FDH) and hydrogenase, converts formic acid into CO_2_ and H_2_ gases (26, 43). Whereas *V. natriegens* has FDH protein homologs, *V. natriegens* does not have hydrogenase genes. In agreement with this bioinformatic result, no H_2_ was detected in *V. natriegens* fermentative cultures by gas chromatography (Fig. 3B).

To deal with anion accumulation from organic acids re-entering the cell, *E. coli* can accumulate K^+^ (31). *V. natriegens* has one homolog to an *E. coli* TrkG or TrkH K^+^ transporter (Table 1). However, it only has a protein with homology for one subunit of an ATP-dependent K^+^ transporter (Table 1); *V. cholerae* also lacks this transporter (not shown). *V. natriegens* also lacks homologs to several small proteins that respond to acute acid stress in *E. coli* (54) and a homolog to the *V. cholerae* regulatory phosphotransferase *vc1080*, which while not an acid tolerance protein itself, is downregulated to avoid formic acid toxicity (55).

*V. natriegens* also lacks amino acid decarboxylases that can eliminate cytoplasmic protons in both *E. coli* and *V. cholerae* (23, 25, 27, 28), but it has one of two H^+^/Cl^-^ antiporters that help prevent hyperpolarization of the membrane during the use of amino acid decarboxylases (56, 57). Amino acid decarboxylases for acid resistance are distinct from anabolism and require an external source of amino acids. We would thus not expect amino acid decarboxylases to be effective in our minimal medium that did not contain amino acids.

**Fig 7:**
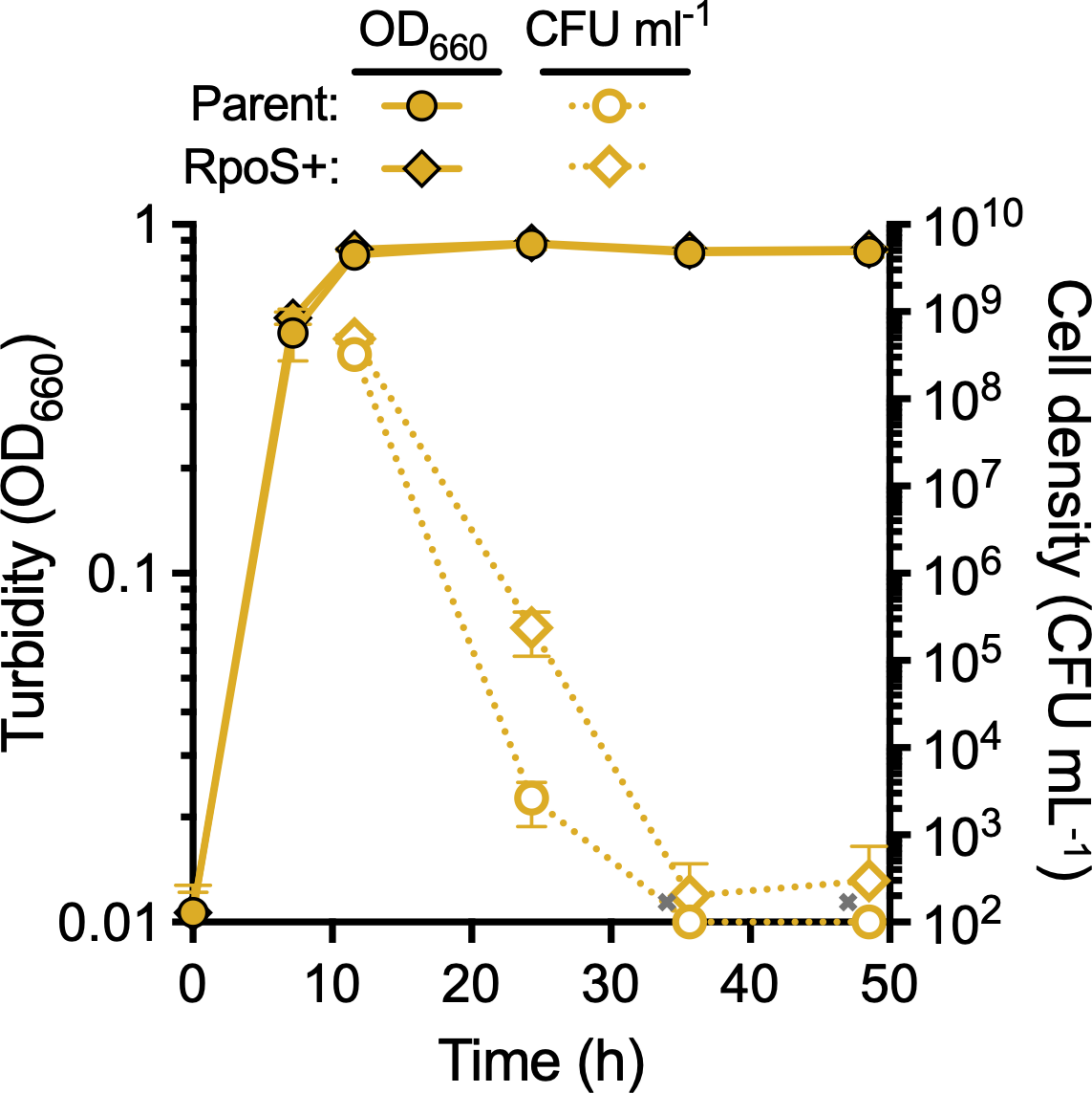
RpoS plays a minor role in *V. natriegens* acid tolerance. Culture turbidity (solid lines; OD_660_) was tracked in minimal media with 25 mM glucose over the entire experiment, whereas viable cell density (dotted lines; CFU mL^−1^) was tracked from the end of the growth phase (~12 h). Data points, mean; error bars, SD; *n* = 3. Some error bars are smaller than the data point symbols. Symbols with an “x” were below the detection limit of 10^2^ CFU mL^−1^.

Amino acid decarboxylases can be regulated by a general stress response sigma factor RpoS in some pathogenic *E. coli* (25). Our *V. natriegens* strain has an *rpoS* mutation causing a premature stop codon (RpoS^E71*^). Given that the RpoS regulon is broad, we decided to investigate if the mutation affects *V. natriegens* acid tolerance in other ways. We thus repaired the stop codon to create an RpoS+ strain. We then compared the survival of the RpoS+ strain to that of the parent after entering stationary phase under fermentative conditions. The decline in RpoS+ viability was still severe upon reaching stationary phase, but the death rate was less pronounced than the parent strain, and the RpoS+ population did not reach extinction within 50 h (100–1,000 CFU mL^−1^; Fig. 7). Thus, RpoS likely plays a role in *V. natriegens* acid tolerance but cannot achieve the same level of acid tolerance as *E. coli*.

### Lactic acid production prolongs growth and metabolism but does not improve survival

**Fig 8:**
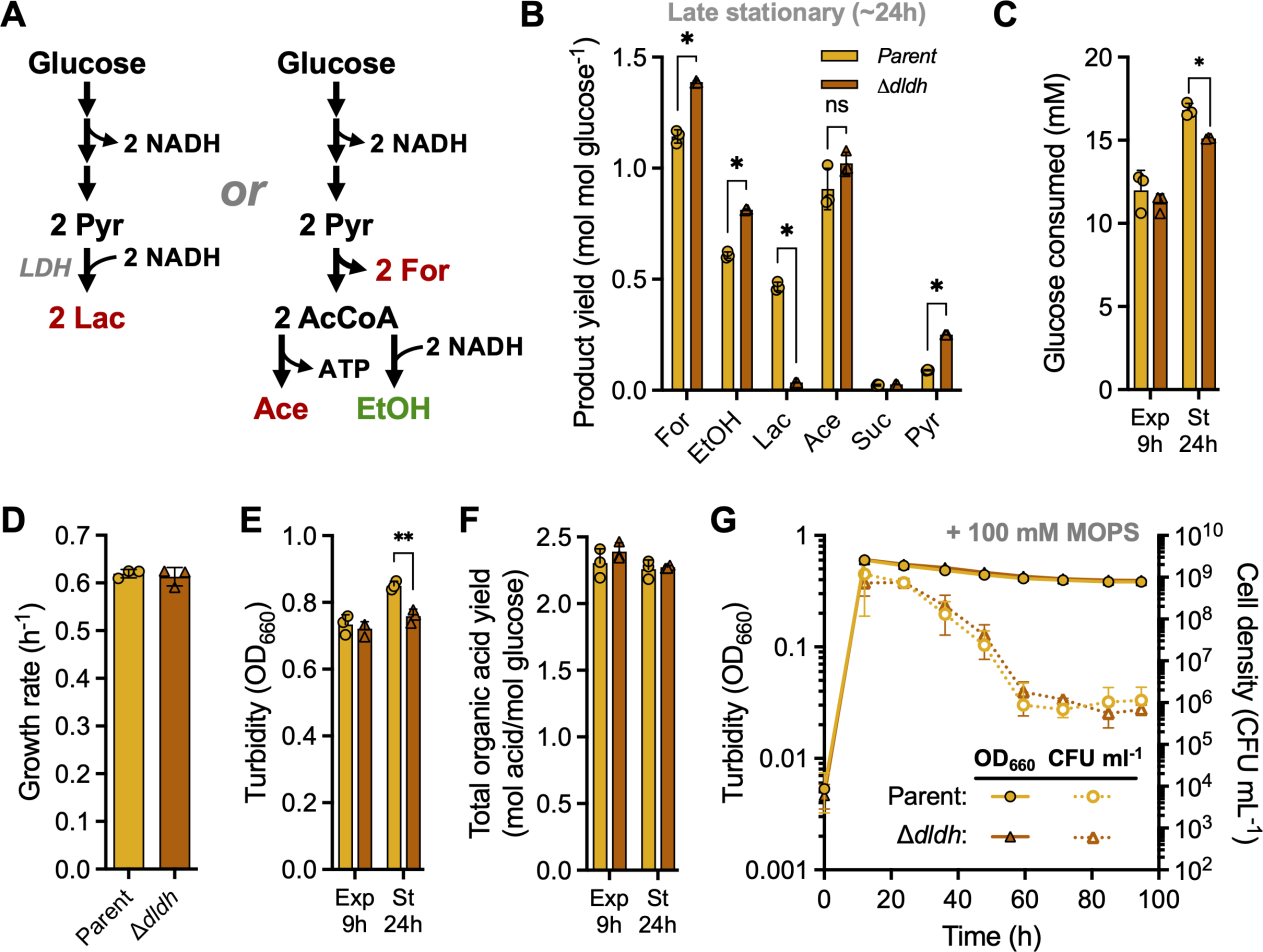
*V. natriegens* LDH extends fermentative metabolism but not viability. (**A**) Lactic acid production can hypothetically achieve electron balance while producing less acid (two lactic acid; left) via LDH compared to producing one ethanol, two formic acid, and one acetic acid (right). Comparison of late stationary-phase (24 h) fermentation product yields (**B**), glucose consumed (**C**), exponential growth rate (**D**), highest cell density measured as turbidity (**E**), and total organic acid yield (**F**) between the parent strain (WT Δ*dns*::Spec^R^) and the LDH mutant (Δ*dldh Δdns::Spec^R^*) in a minimal medium with 25 mM glucose without MOPS. For, formic acid; EtOH, ethanol; Lac, lactic acid; Ace, acetic acid; Suc, succinic acid; Pyr, pyruvate. (**C, E, F**) Exponential phase samples (9 h, Exp); Stationary-phase samples (24 h, St). (**G**) Turbidity (solid lines; OD_660_) and viable cell density (dotted lines; CFU mL^−1^) were tracked into stationary phase in a minimal medium with 25 mM glucose plus 100 mM MOPS. Data points, mean; error bars, SD; *n* = 3. The detection limit is 10^2^ CFU mL^−1^. (**B–F**) Points, biological replicate values; error bars, SD; *n* = 3. Statistical differences were determined using unpaired, two-tailed *t*-tests; ns, not significant; *, *P* < 0.05; **, *P* < 0.01.

Organisms that produce multiple fermentation products can alter their fermentation profile to respond to environmental conditions. Unlike *V. cholerae*, *V. natriegens* lacks genes for producing neutral fermentation products (Table 1) (24). However, like *E. coli*, *V. natriegens* produced lactic acid in late exponential phase and into stationary phase (Fig. 2A and B). In *E. coli*, cytoplasmic lactate dehydrogenase (LDH) expression increases in response to acidic pH (29), suggesting that lactic acid production might decrease acid stress (30). A shift to lactic acid production over acetic acid production was also correlated with enhanced survival of pathogenic *E. coli* O157:H7 over an *E. coli* K-12 strain, likely by avoiding accumulation of cytoplasmic acetate anions (31). Shifting to lactic acid production would result in less ATP but also less acid overall to maintain electron balance (Fig. 8A) ; two pyruvate could either go to two lactic acid (pKa, 3.86) or to one ethanol + 2 formic acid (pKa, 3.75) + 1 acetate (pKa, 4.76). The latter pathway would generate more organic acids, one of which is acetic acid that would protonate more readily at low pH due to its high pKa. Acetic acid/acetate is also potentially more toxic than lactic acid/lactate in general (32).

To test whether lactic acid production contributes to *V. natriegens* survival under fermentative conditions, we first created an LDH mutant. *V. natriegens* has three putative LDH genes, PN96_RS20515 (WP_020334824), PN96_RS17000 (*dldh*; WP_020334627), and PN96_RS17015 (*lldh*; WP_014233519). The latter two genes had been deleted by another group and had little effect on fermentative lactic acid production (10). However, the conditions in that study favored succinic acid production over lactic acid production, whereas we observed the opposite. Using the fermentative LDH gene from *E. coli* MG1655 (b1380) as a BLASTp query, we hypothesized that *dldh* was most likely the *V. natriegens* fermentative LDH (100% coverage, 62% identity); the other two candidates did not show significant sequence identity to the query sequence. Indeed, when we deleted *V. natriegens dldh*, the lactic acid yield was close to zero at 24 h (Fig. 8B). Compared to the parent strain, the Δ*dldh* mutant produced 1.2 times more formic acid and 1.3 times more ethanol (Fig. 8B), a route that would replace the electron balancing role of lactic acid production (Fig. 8A). The Δ*dldh* mutant also produced 2.8 times more pyruvate, suggesting that a bottleneck was created in the absence of LDH. The Δ*dldh* mutant consumed less glucose than the parent (Fig. 8C), which did not affect the growth rate (Fig. 8D), but the final Δ*dldh* mutant turbidity was 90% of that of the parent (Fig. 8E). Still, the total organic acid yield was not significantly different between the two strains, suggesting that even without lactic acid production, *V. natriegens* faced comparable acid exposure (Fig. 8F).

We thus examined the effect of the LDH deletion on *V. natriegens* stationary-phase survival under fermentative conditions. Anticipating that the rapid death rate would make a comparative analysis difficult, we added 100 mM MOPS to slow the death rate. Stationary-phase survival was similar between the parent and Δ*dldh* strains (Fig. 8G). Thus, whereas, in the absence of MOPS, lactic acid production extends glucose consumption into stationary phase (Fig. 8C) and potentially allows for higher cell densities, at least as measured by turbidity (Fig. 8E), it does not improve stationary-phase survival under fermentative conditions with MOPS, wherein wild-type lactate production is elevated over other fermentation products (Fig. 6C).

Below we speculate why lactic acid might be produced during the transition out of exponential growth and into stationary phase. One possibility is that acetate and ethanol production becomes less available as an option. For example, as the pH drops and growth slows, the biosynthetic demand for ATP would also slow. This might limit ADP availability for acetate production and create a bottleneck at pyruvate (Fig. 8A). LDH offers a carbon and electron sink to maintain metabolic flow and help alleviate this bottleneck; a role for LDH in addressing this bottleneck would also explain the elevated pyruvate excretion in the Δ*dldh* mutant (Fig. 8B). If we assume similar kinetic parameters to *E. coli* enzymes, it is possible LDH is poised to respond to pyruvate accumulation due to its increased expression and improved half-saturation constant for pyruvate relative to PFL at low pH (58–60). Characterization of the kinetic properties of these *V. natriegens* enzymes is needed to test this hypothesis.

### Conclusion

As interest grows in *V. natriegens* as a bioprocessing strain, one must weigh its favorable attributes of rapid growth and genetic tractability against its less favorable attributes. Here we report that, relative to *E. coli*, *V. natriegens* is comparatively sensitive to the acidic conditions that arise during mixed acid fermentations. This difference in acid tolerance is likely due to *V. natriegens* lacking most of the acid resistance mechanisms that have been characterized in *E. coli*. Such acid-resistance mechanisms could potentially be engineered to improve *V. natriegens* acid tolerance. Increasing the buffering capacity of the fermentation medium would also avert acid sensitivity, but one would need to weigh the associated cost under industrial conditions. Thus, acid sensitivity will need to be considered when engineering *V. natriegens* for bioprocessing, unless the goal is to engineer *V. natriegens* to produce neutral products.

## MATERIALS AND METHODS

### Strains

Experiments used wild-type *E. coli* MG1655 (61) and the *Vibrio natriegens* ATCC 14048 (12) variant “Dalia, SAD1302, 2016,” which carries the following mutations: RpoS^E71*^, PN96_RS14445^S29P^ (putative cation solute symporter); Cultivarium Portal (https://portal.cultivarium.org/communities/vnat-sequencing?tab=data&dataset=Pilot%20study). *V. natriegens* mutants were made as described (12), except the transformation mixtures of cells and PCR products were incubated statically at 30°C for 12–16 h before outgrowth, and selection plates used 250 µg spectinomycin mL^−1^. The *V. natriegens* Δ*dldh* and RpoS+ strains were made in *V. natriegens* TND1964, which is the wild-type strain with pMMB-*tfoX* that allows for IPTG-inducible competency (12). A Δ*dldh* (ΔRS17000) construct was made by PCR amplifying (Q5 DNA polymerase; New England Biolabs) 3 kb upstream and downstream fragments. The two fragments were then combined by splicing-by-overlap extension PCR using the outermost primers. The RpoS+ construct was made using gDNA from a *V. natriegens* strain wherein *rpoS* was already repaired (Glasgo and Zinser, manuscript in prep). Each construct (1 µg) was then co-transformed into TND1964 with 50 ng of a DNA fragment containing a spectinomycin resistance cassette flanked by ~1 kb of homology to the upstream and downstream regions of the *dns* locus (PN96_RS00885) amplified from *V. natriegens* SAD1306 genomic DNA (12). The “parent” strain (NWH004) used as the control strain in experiments with the Δ*dldh* mutant was derived from TND1964 by introducing a spectinomycin resistance cassette at the *dns* locus. Colonies were screened for RpoS activity by a positive colony bubble test using 30% H_2_O_2_ (62). Colonies were screened for Δ*dldh* by PCR using OneTaq DNA polymerase (New England Biolabs). All mutations were confirmed by Sanger sequencing. Primers are listed in Table 2.

**TABLE 2:** Primers

Primer	Sequence (5′→ 3′)^*a*^	Description^*b*^
NH022	CGAGGTGAAGATCATTCATTTCC	F for amplifying Δ*dns::Spec^R^*
NH025	CTTAGTGATTGGGTCACTCATTGG	R for amplifying Δ*dns::Spec^R^*
NH014	CTCTGCACCACTACCGTC	F for confirming *dns*
NH015	CGAATACCGATGTCGCTGC	R for confirming *dns*
NH074	CAGGTATAGCCTTTGATAGAAGG	Upstream F for Δ*dldh*
NH075	cgagagagaaccatgATCAACTAAATAAGGAATCGAGGAGG	Upstream R for Δ*dldh*
NH076	ccttatttagttgatCATGGTTCTCTCTCGAAATCATTG	Downstream F for Δ*dldh*
NH077	CCATGTATTGCAGAGCACTC	Downstream R for Δ*dldh*
NH078	CGCTCTTTGTTAAGCTCATAAC	F for confirming Δ*dldh*
NH079	CGAACGCACAACATACAAG	R for confirming Δ*dldh*
NH238	GATGGGAATAACCTAGAAGAAGC	F for amplifying *rpoS*
NH239	GCCATTGTTGATTATCTGCG	R for amplifying *rpoS*
NH240	CTTGCTAACTCGCCAGGG	F for confirming *rpoS*
NH241	GTGCTTTTTCACCATCACCAC	R for confirming *rpoS*

^*a*^ Lowercase indicates overlapping regions for SOE PCR.

^*b*^ F, forward; R, reverse.

### Growth conditions

Colonies were grown from 25% glycerol frozen stocks on agar plates of either lysogeny broth Miller medium (LB; *E. coli*), or LB supplemented with an additional 20 g NaCl L^−1^ (LB3; *V. natriegens*). LB3 was supplemented with either carbenicillin (100 µg mL^−1^), spectinomycin (250 µg mL^−1^), or 100 µM IPTG when appropriate. LB3 was also used for strain construction.

For all other experiments, anoxic minimal M9-derived coculture medium (MDC) (63) for *E. coli* or MDC with an additional 80 mM NaCl for *V. natriegens* (MDC80) was prepared by aliquoting 10 mL media into 27-mL anaerobic tubes or 60 mL media into 150-mL serum vials, bubbling with N_2_, then sealing with rubber stoppers and aluminum crimps before autoclaving. Media supplements were then added by syringe to the following final concentrations from anoxic 100× stock solutions: glucose, 25 mM; NH_4_Cl, 10mM; MgSO_4_, 1 mM; CaCl_2_, 0.1 mM. A 2 M MOPS solution was prepared by dissolving the free acid in ultrapure water and adjusting the pH to 7.0 using NaOH pellets. To make an anoxic stock, the solution was then filter-sterilized into a serum vial and flushed with filter-sterilized argon. This solution was added to cultures to the indicated concentration after autoclaving, with sterile ultrapure water also added where necessary to have common dilution effects between cultures. Starter cultures were inoculated into MDC or MDC80 from single colonies. Late-exponential phase starter cultures (0.65–0.85 OD_660_) were then used to inoculate test media to an initial cell density of ~0.01 OD_660_. Tubes were incubated horizontally at either 30°C (*V. natriegens*) or 37°C (*E. coli*) with shaking at 150 rpm (¾” stroke length).

### Analytical procedures

A Genesys 20 spectrophotometer (Thermo Fisher) was used to measure cell density as turbidity at 660 nm (OD_660_) either directly in 27-mL culture tubes or in 1-mL samples in cuvettes when 150-mL serum vials were used; we chose 660 nm to be consistent with our previous studies involving *V. natriegens* and *E. coli* (38, 44). Growth rates were determined by fitting an exponential trendline to turbidity readings taken between 0.1 and 0.8 OD_660_ using GraphPad Prism v.6. Turbidity measurements were converted into molar biomass using a conversion factor of 270 (*V. natriegens* [64]) or 300 (*E. coli* [65, 66]) mg DCW L^−1^ OD_600_^−1^ and dividing by a molecular weight of 26.21 mg mmol^-1^ (*V. natriegens* [67]) or 24.97 (*E. coli* [68]) based on elemental compositions (67, 68).

Glucose and fermentation products were quantified in supernatant samples by HPLC (Shimadzu) as described (69). Temporal assessment of fermentations was performed in 60-mL cultures in 150-mL serum vials. Samples (1 mL) were taken at regular intervals; air contamination was minimized by flushing a 1-mL syringe and needle with filter-sterilized N_2_ before sampling. To quantify H_2_, 0.1 mL of headspace was sampled using a gas-tight syringe and injected into a Shimadzu GC-2014 gas chromatograph with a thermal conductivity detector as described (70). The H_2_ limit of detection was 12 nmol. To measure pH, cultures were centrifuged, and supernatants were assayed using a pH meter.

Viable cell counts were quantified as CFUs using the track plate method (71). Samples (0.4–0.5 mL) were removed from 10-mL cultures using an N_2_-flushed syringe and serially diluted in oxic MDC or MDC80 in a 96-well plate. The last six dilutions were spotted (10 µL) onto LB or LB3 agar using a multichannel pipette. A minimum of 1 colony was used as a cutoff, making the limit of detection 100 CFU mL^−1^ for an undiluted sample.

For experiments using nongrowing cells, late-exponential phase cultures (0.65–0.85 OD_660_) were inoculated into anoxic MDC or MDC80 with all supplements except glucose. The target inoculum was 10^7^ cells mL^−1^. Specified volumes of a solution containing 1 M acetic acid plus 1 M formic acid was then added by syringe. Cell suspensions were then sampled for CFU measurements. Death rates were determined from the slope of a semi-log equation (linear X, log Y) fitted to experimental data using GraphPad Prism v.6.

### Bioinformatics

NCBI’s BLASTp (47) was used to identify putative acid-resistance genes in *V. natriegens* ATCC14048 (NCBI RefSeq: GCF_001456255.1) using amino acid sequences from *E. coli* MG1655 (NCBI RefSeq: GCF_000005845.2) or *V. cholerae* O1 biovar El Tor str. N16961 (GCF_000006745.1). *V. natriegens* proteins that had >25% identity with >50% query coverage were considered putative homologs.

### Statistics

GraphPad Prism v.6 was used for all statistical analyses.
